# Matricellular Signal Transduction Involving Calmodulin in the Social Amoebozoan *Dictyostelium*

**DOI:** 10.3390/genes4010033

**Published:** 2013-02-15

**Authors:** Danton H. O’Day, Robert J. Huber

**Affiliations:** 1 Department of Biology, University of Toronto Mississauga, 3359 Mississauga Road North, Mississauga, ON, L5L 1C6, Canada; 2 Department of Cell and Systems Biology, University of Toronto, 25 Harbord Street, Toronto, ON, M5S 3G5, Canada; 3 Center for Human Genetic Research, Massachusetts General Hospital, Harvard Medical School, Richard B. Simches Research Center, 185 Cambridge Street, Boston, MA 02114, USA; E-Mail: rhuber@chgr.mgh.harvard.edu

**Keywords:** matricellular, signal transduction, EGF-like repeats, extracellular matrix, calmodulin, morphogenesis, cell differentiation, development

## Abstract

The social amoebozoan *Dictyostelium discoideum* undergoes a developmental sequence wherein an extracellular matrix (ECM) sheath surrounds a group of differentiating cells. This sheath is comprised of proteins and carbohydrates, like the ECM of mammalian tissues. One of the characterized ECM proteins is the cysteine-rich, EGF-like (EGFL) repeat-containing, calmodulin (CaM)-binding protein (CaMBP) CyrA. The first EGFL repeat of CyrA increases the rate of random cell motility and cyclic AMP-mediated chemotaxis. Processing of full-length CyrA (~63 kDa) releases two major EGFL repeat-containing fragments (~45 kDa and ~40 kDa) in an event that is developmentally regulated. Evidence for an EGFL repeat receptor also exists and downstream intracellular signaling pathways involving CaM, Ras, protein kinase A and vinculin B phosphorylation have been characterized. In total, these results identify CyrA as a true matricellular protein comparable in function to tenascin C and other matricellular proteins from mammalian cells. Insight into the regulation and processing of CyrA has also been revealed. CyrA is the first identified extracellular CaMBP in this eukaryotic microbe. In keeping with this, extracellular CaM (extCaM) has been shown to be present in the ECM sheath where it binds to CyrA and inhibits its cleavage to release the 45 kDa and 40 kDa EGFL repeat-containing fragments. The presence of extCaM and its role in regulating a matricellular protein during morphogenesis extends our understanding of CaM-mediated signal transduction in eukaryotes.

## 1. Introduction

### 1.1. Matricellular Protein-Mediated Signal Transduction

The matricellular protein component of the extracellular matrix (ECM) functions as a modulator and mediator of cell-matrix interactions [1,2,3]. Unlike collagens and laminins, they do not contribute to the physical properties or organization of extracellular structures. These proteins demonstrate several attributes: they function as both soluble and insoluble ECM components, they associate with extracellular proteases and growth factors, and they are expressed at high levels during developmental events. Among other characteristics, matricellular proteins modulate cellular processes by binding to cell surface receptors and initiating intracellular signal transduction. The most well characterized matricellular proteins are SPARC (secreted protein acidic and rich in cysteine), tenascin cytotactin (tenascin C) and thrombospondin (TSP) 1 and 2 [2,4,5]. Metalloproteases of the ADAMTS (a disintegrin and metalloproteinase with thrombospondin motifs) superfamily of proteins cleave matricellular proteins, including thrombospondins, resulting in the extracellular release of small signaling polypeptides and peptides [6]. 

Epidermal growth factor-like (EGFL) repeats are a common feature of cysteine-rich matricellular proteins [2]. EGFL repeats, which can be present as single entities or as multiple tandem repeats, are a widespread but highly variable domain containing cysteine residues reflecting the position of these residues in EGF [7,8]. While they have been well studied in certain human proteins they are also present in lower eukaryotes, including the model organisms *Drosophila melanogaster* and *Dictyostelium discoideum* [9,10,11].

Tenascin C, TSP-1, and laminin-5 are the best studied EGFL repeat-containing mammalian ECM proteins. The EGFL repeats of these proteins initiate intracellular signal transduction events that modulate cell movement. For example, the EGFL repeats of tenascin C (esp. Ten14) increase the rate of cell motility by binding to the EGFR and activating EGFR-dependent signaling [12,13]. Ten14 functions at micromolar concentrations but, unlike EGF, binding of Ten14 is transient and does not lead to internalization [13,14]. Due to its transient binding, Ten14 can mediate continuous activation of the receptor and thus sustain the increased cell motility induced by the EGFL repeat. The EGFL repeats of TSP-1 also increase the rate of cell movement by activating intracellular signaling events [15]. TSP-1 EGFL repeats do induce autophosphorylation of the EGFR but not by binding to the receptor, suggesting that not all EGFL repeats bind to the EGFR to enhance cell movement [15]. While not a true matricellular protein, laminin-5 does possess EGFL repeats which increase the rate of cell movement by binding to the EGFR [16]. When cleaved by matrix metalloproteinase 2 (MMP2), the resulting EGFL repeat-containing cleavage products activate the EGFR and downstream signaling pathways. Overall, studies on these proteins strongly suggest that a primary function of cysteine-rich, EGFL repeats present within ECM proteins is to regulate cell movement. The results for *Dictyostelium* suggest this function may be evolutionarily conserved.

### 1.2. ECM Proteins of Dictyostelium discoideum

*Dictyostelium*, a eukaryotic social amoebozoan, is a widely used model biological system for studying cellular and developmental processes [17]. When single cells are starved they enter a developmental program that begins with cell aggregation to produce multicellular tissue-like aggregates. The aggregation process is driven by cyclic AMP (cAMP)-mediated chemotaxis, an event that has been extensively studied and reviewed [18,19,20]. The resulting aggregates develop into multicellular pseudoplasmodia or slugs that exhibit a pattern and polarity of contained prespore and prestalk cells. The cells of the slug are surrounded by an ECM historically referred to as a slime sheath. This sheath, which is continuously synthesized from the tip (front) of the slug, was considered to serve as protection against desiccation [21]. Under appropriate conditions, the slugs culminate into fruiting bodies comprised of dead stalk cells supporting a mass of viable spores [22]. 

The *Dictyostelium* sheath shares similarity in structure and composition to the ECMs of both animals and plants. It is made up of cellulose and other polysaccharides embedded in a matrix of structural and non-structural proteins. Glycoproteins called sheathins (*i.e.*, EcmC, EcmD, EcmE) co-localize with cellulose and are involved in regulating slug migration [23]. EcmA is a well-studied sheath protein that has been shown to be an integral structural protein distributed throughout the ECM. However, EcmA gene knockout (*ecmA-*) cells still form slugs that migrate normally in spite of a weakening of their ECM [24]. Other work has identified a group of soluble, mobile glycoproteins that localize within the sheath but their identity has not been determined [25,26]. More recently, the protein CyrA has been identified as another ECM component in *Dictyostelium* [27,28]. 

## 2. Current Research

### 2.1. CyrA is a CaM-binding EGFL Repeat-Containing ECM Protein

**Figure 1:**
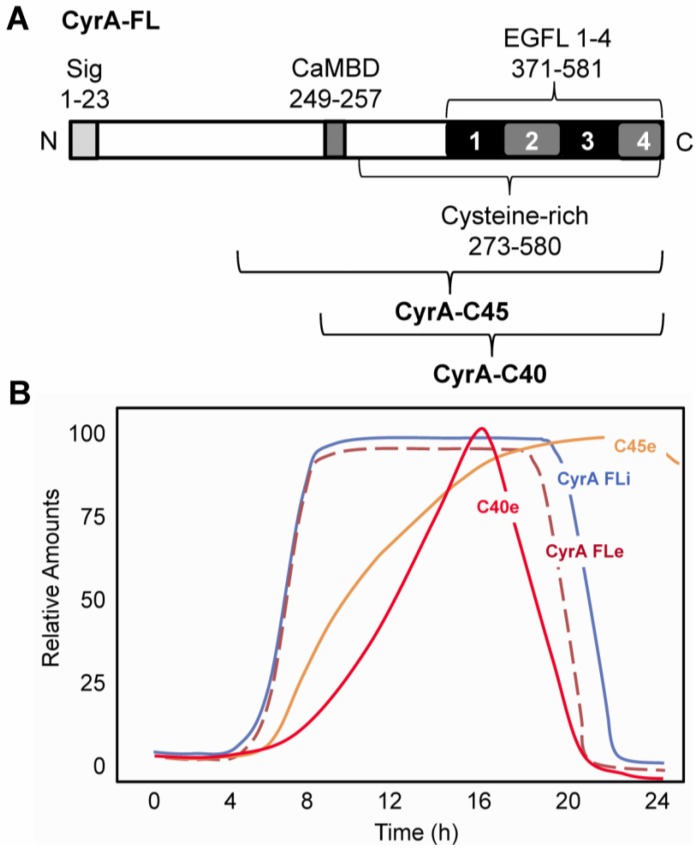
The structure and processing of CyrA. A. CyrA possesses an N-terminal signal sequence (Sig), a calmodulin-binding domain (CaMBD) and four tandem EGF-like repeats (EGFL1-4) in its cysteine-rich C-terminal region. The presumptive C-terminal CyrA-C45 and CyrA-C40 proteolytic products are shown. B. Time course of accumulation of full-length intracellular (CyrA FLi) and extracellular (CyrA FLe) CyrA and extracellular proteolytic products CyrA-C45 (C45e) and CyrA-C40 (C40e).

A cDNA encoding CyrA, a novel, cysteine-rich, putative calmodulin (CaM)-binding protein (CaMBP) was isolated using the CaM-binding overlay technique [27,29]. CyrA is characterized by the presence of a signal sequence (i.e., target for secretion), a CaM-binding domain (CaMBD) and four tandem EGFL repeats (EGFL1-4) that comprise the C-terminal region of the protein (Figure 1, A). These repeats, especially EGFL1, show strong sequence similarity to Ten14 [27]. CyrA, which binds to CaM both in the presence and absence of calcium ions (Ca^2+^), is secreted during growth and development [27]. In keeping with it being a secreted protein, both CyrA immunolocalization and CyrA-GFP localization showed that the protein localizes to the endoplasmic reticulum, particularly its perinuclear component [28]. Western blot analyses revealed that the intracellular expression of full-length CyrA (~63kDa) peaks between 12 and 16 hours of development, the time when multicellular slug formation occurs (Figure 1, B) [28]. At this time, CyrA is secreted at very high levels and localizes to the ECM (*i.e*., sheath) of the migrating slug. Immunolocalization of CyrA revealed that it is predominantly localized at the tip of the slug with decreasing abundance towards the back [27]. In various experiments cells were treated with pharmacological agents: LY294002 (PI3K inhibitor), quinacrine (PLA2 inhibitor), W7 (CaM inhibitor), and TMB-8 (intracellular calcium release inhibitor). Pharmacological intervention yielded insight into the regulation of CyrA secretion by showing that it was dependent on intracellular Ca^2+^ release as well as active CaM, phosphatidylinositol 3 kinase (PI3K), and phospholipase A2 (PLA2) function [28].

During development CyrA is secreted and proteolytically cleaved to release two major extracellular C-terminal fragments of approximately 45kDa and 40kDa (Figure 1, B) [27]. PEST sequences are rich in proline (P), glutamic acid (E), serine (S), and threonine (T) and are considered to act as signal peptides for protein degradation. The size of these fragments and the location of the putative PEST (331-388) sequence indicate they would contain all four of the EGFL repeats. *Dictyostelium* secretes a large number of proteases but the CyrA cleaving protease has not yet been identified [30]. Like the EGFL repeat-containing cleavage products from mammalian matricellular proteins, EGFL1 of CyrA enhances the rate of cell motility in *Dictyostelium* [31,32]. 

### 2.2. EGFL1 Peptide Increases Cell Motility and Chemotaxis

Treatment of *Dictyostelium* cells with a peptide of identical sequence to the first 18 amino acids of EGFL1 (DdEGFL1) results in a 2-6-fold increase in random cell motility and an 85% increase in cAMP-mediated chemotaxis, depending on the strain used [31,32]. The over-expression of CyrA also increases the rate of cAMP-mediated chemotaxis, providing *in vivo in vivo* support for the role of CyrA as a normal mediator of cell movement and cAMP chemotaxis in *Dictyostelium* [28]. EGFL1 is not a chemoattractant for *Dictyostelium* cells, however it does activate signaling pathways that function in a supportive role to increase the rate of both random cell movement and cAMP chemotaxis during development [33]. As such, starvation increases the response of cells to EGFL1 [27]. The localization of CyrA to the ECM during multicellular development supports the concept that EGFL1 is involved in regulating the movement of cells within the slug during its movement and during morphogenesis [27,28,34]. 

### 2.3. EGFL1-Mediated Signal Transduction

**Figure 2:**
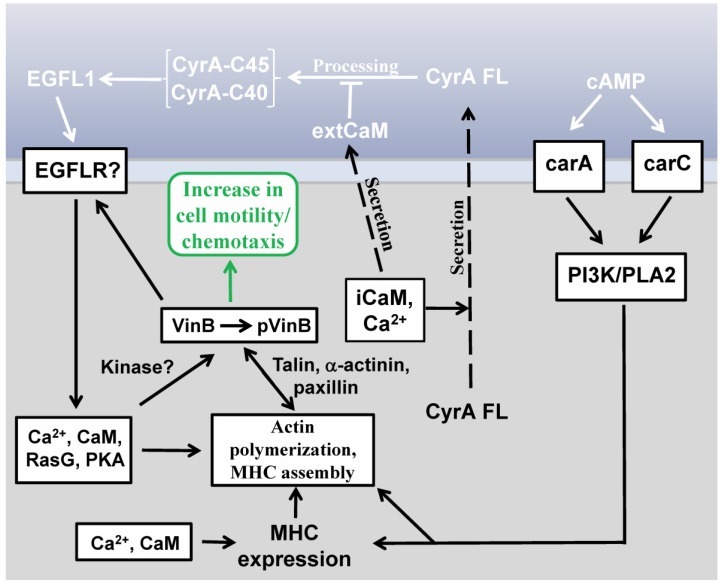
The primary signal transduction events mediating the increase in cell motility and chemotaxis induced by EGFL1. Secretion of full-length CyrA (CyrA FL) is Ca^2+^ and calmodulin (CaM) dependent. Extracellularly CyrA FL becomes part of the extracellular matrix where it is processed releasing two smaller EGFL repeat-containing C-terminal fragments of ~45 kDa (CyrA-C45) and ~40 kDa (CyrA-C40). This proteolytic cleavage is inhibited by extracellular CaM (extCaM). At least one EGFL repeat (*i.e.*, EGFL1) within the 45kDa and 40kDa CyrA fragments binds to an unidentified receptor to activate intracellular signaling events which will ultimately increase both random cell motility and chemotaxis (rounded box). As detailed in the main text, these signaling events involve kinase signaling (e.g., PKA), Ca^2+^, CaM, and Ras G which oversee actin polymerization and myosin heavy chain (MHC) assembly. For cells responding chemotactically to cAMP via carA or carC, stimulation of the PI3K/PLA2 signaling pathways increases MHC expression, MHC assembly and actin polymerization. MHC expression is also regulated by Ca^2+^ and intracellular CaM (iCaM). Ca^2+^, iCaM, Ras G plus an unidentified kinase also are involved in the sustained phosphorylation of vinculin B-related (VinB) induced by EGFL1. Phosphorylated VinB (pVinB) working in conjunction with binding proteins talin, α-actinin and paxillin along with the actin cytoskeleton, leads to increased cell motility and chemotaxis. The details of each step in the figure are covered in the main text of this review.

A model of the major signaling events mediated by EGFL1 is presented in Figure 2. EGFL1 increases the rate of *Dictyostelium* random cell movement via a novel signaling pathway that does not require signaling mediated by either of the two cAMP receptors that are active during early development (carA and carC) [32]. The increase in random cell movement induced by EGFL1 requires signaling involving CaM and intracellular Ca^2+^ release and leads to increases in polymeric actin and myosin II heavy chain (MHC) in the cytoskeleton [32]. The cytoskeletal proteins talin B (TalB) and paxillin B (PaxB), which are homologues of mammalian talin and paxillin, respectively, are also involved in translating the EGFL1 signal into an increase in random cell motility [35]. While the activities of both PI3K and PLA2, two signaling proteins that mediate the chemotaxis of *Dictyostelium* amoebae in parallel compensatory pathways, are required for EGFL1-enhanced random cell motility, PLA2 appears to be the more dominant regulator [31,33].

Both Ca^2+^ and Ras signaling are required for EGF-induced cell movement in normal mammalian and cancer cells [36,37]. In *Dictyostelium*, RasC and RasG have been shown to regulate chemotaxis towards cAMP and EGFL1-increased movement partially requires the activity of RasG, but not RasC [32]. The cAMP-dependent serine/threonine kinase, protein kinase A (PKA), which is involved in regulating cell movement and cAMP chemotaxis in *Dictyostelium*, is also required for the increased rate of cell movement induced by EGFL1 function showing that PKA kinase activity is required for EGFL1 signal transduction [35,38]. In keeping with this, EGFL1-induced phosphorylation of a 90 kDa phosphotyrosine protein during cell starvation requires PKA activity [35]. Other as yet unidentified kinases also are involved in EGFL1 signaling since two unidentified phosphotyrosine proteins appear in DdEGFL1 pull-down assays [35].

Early work on cell motility in *Dictyostelium* revealed that random movement occurs in the absence of functional heterotrimeric G proteins but instead PI3K and Ras mediate this Gβ-independent signaling pathway [39,40]. It was suggested that during cAMP chemotaxis the Gβ-dependent pathway takes over from the Gβ-independent pathway. The work done on EGFL1 signal transduction reveals that activation of the Gβ-independent signaling pathway can be induced by EGFL repeat-containing proteins providing more understanding of how cell motility is regulated in this social amoebozoan [32]. As discussed in the next section, insight into the role of one cytoskeletal component is an example of this.

### 2.4. Vin B Phosphorylation is Regulated by EGFL1 Signal Transduction

During the starvation of *Dictyostelium* cells a 210 kDa protein is dephosphorylated [31]. Addition of EGFL1 peptide (*i.e.*, DdEGFL1) to starved cells sustains the threonine phosphorylation of this protein. To identify the protein, immunoprecipitation coupled with an LC/MS/MS analysis was carried out revealing it to be vinculin B (VinB) [35]. Since VinB shares sequence similarity with mammalian vinculin, a protein that links the actin cytoskeleton to the plasma membrane, its potential co-localization with the cytoskeleton was investigated. Threonine phosphorylated VinB (pVinB) as well as VinB-GFP both localized to the cytoplasm of *Dictyostelium* amoebae with specific localization to the cytoskeleton [35]. Furthermore, VinB-GFP undergoes threonine phosphorylation and co-immunoprecipitates with the known vinculin-binding cytoskeletal proteins MHC, actin, alpha-actinin, and talin [35]. 

**Figure 3:**
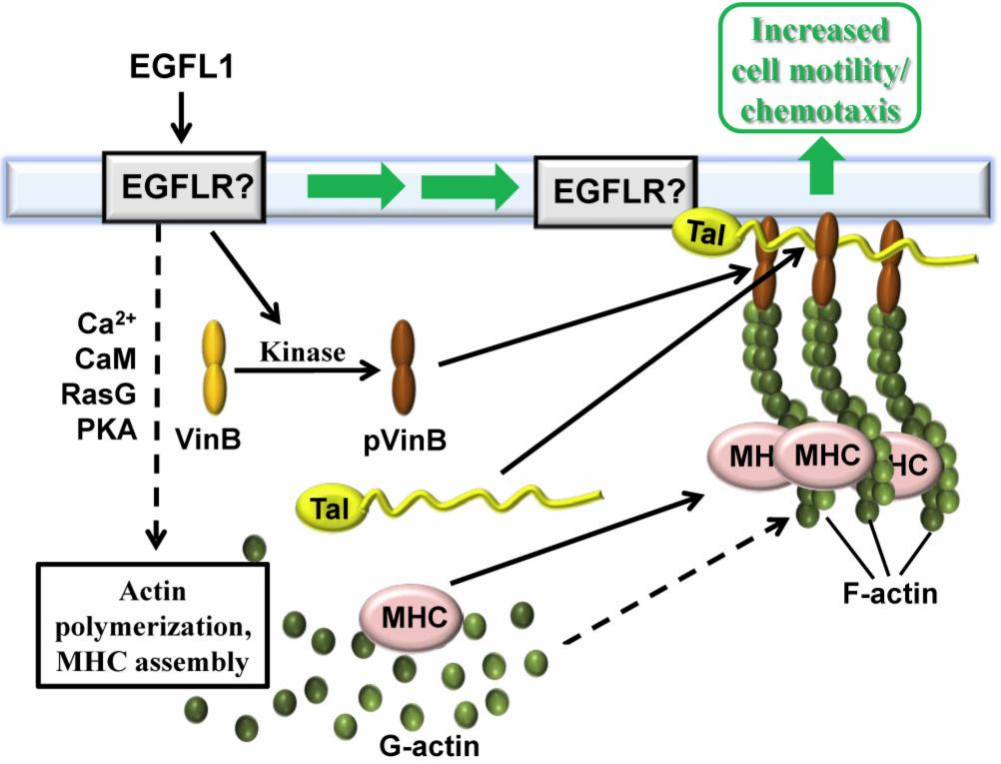
Involvement of vinculin B in EGFL repeat-enhanced cell movement. EGFL1 binds to an uncharacterized receptor to initiate intracellular signal transduction involving Ca^2+^, CaM, RasG and PKA. These events underlie the increased expression and assembly of myosin II heavy chain (MHC) as well as the polymerization of actin. Concomitant with this, vinculin B (VinB) is threonine phosphorylated by signal transduction (pVinB) involving an uncharacterized kinase leading to its association with talin, actin, and MHC to mediate the increased cell movement that is induced by EGFL1 binding.

Mutant analysis further revealed that EGFL1-increased cell movement requires the cytoskeletal-associated proteins TalB and PaxB [35]. The threonine phosphorylation of VinB is independent of PI3K/PLA2 signaling and PKA kinase activity [35]. In addition to revealing aspects of the function of VinB, this work also provided insight into the signaling pathways involved in EGFL1 regulated cell movement. A model for the involvement of vinculin B in EGFL repeat-enhanced cell movement is presented in Figure 3.

### 2.5. Extracellular CaM and its Multiple Functions

The identification of CyrA as an extracellular CaMBP at first seemed enigmatic. While the literature abounds with data on the myriad of functions of intracellular CaM, the literature on extracellular CaM (extCaM) has been sporadic and diverse resulting in a lot of skepticism about the validity of its true presence outside of cells and its function there. The evidence for extCaM is strongest in plants where it regulates several functions including cell wall regeneration, gene regulation, germination and proliferation in various species [41,42]. In animals, extCaM mediates DNA synthesis and cell proliferation in a number of species [43,44]. Individual studies have also implicated it in limb regeneration and vasodilation [45,46]. However, no reports for extCaM existed for any eukaryotic microbe.

**Figure 4:**
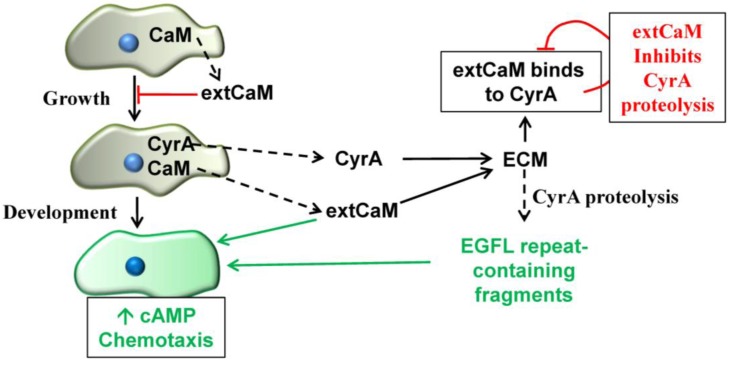
Functions of extracellular calmodulin in *Dictyostelium*. During growth calmodulin (CaM) is secreted into the extracellular medium (extCaM) where it feeds back to inhibit cell proliferation. When cells are starved they embark on multicellular development which begins with cAMP-mediated chemotaxis. ExtCaM alone mediates cAMP chemotaxis. In addition as development progresses, extCaM and secreted CyrA become part of the extracellular matrix (ECM) surrounding the multicellular slug. At this time EGFL1-containing fragments of CyrA increase the rate of cAMP chemotaxis. The binding of extCaM to CyrA inhibits its proteolytic processing reducing the rate of EGFL1-fragment production.

A proteomic analysis of extracellular proteins from growing and developing *Dictyostelium* cells indicated the presence of CaM in the extracellular medium [30]. The existence of extCaM in *Dictyostelium* was validated in subsequent studies on CyrA. Western blotting revealed that CaM is present in the extracellular medium during growth and treatment of growth phase cells with exogenous CaM inhibits cell proliferation [27,47]. While the presence of extCaM during growth was suspected, our analyses unexpectedly revealed that high and constant levels of extCaM are present during growth as well as during starvation, aggregation (*i.e.*, cAMP mediated-chemotaxis), multicellular tissue formation (*i.e.*, slug formation) and later developmental stages [27,47]. The absence of major cytoplasmic proteins (e.g., tubulin) in the extracellular medium verified that this extCaM is due to secretion and not cell death [27]. These events and the multiple functions of extCaM are summarized in Figure 4. When cells starve, as a prelude to multicellular development, extCaM comes into play for the developmental regulation of cAMP chemotaxis that drives cell aggregation [47]. Earlier work showed that antagonizing CaM inhibited cAMP chemotaxis with several CaMBPs directly linked to the event [48]. These results were supported and extended when exogenous CaM was shown to enhance cAMP chemotaxis [47]. Ongoing and continued investigations into the CaMBP CyrA and its EGFL repeats reinforced the role of extCaM and extracellular CaMBPs in this event.

During the later stages of development extCaM is found to be specifically localized in the ECM [28]. The multicellular slug, which is motile, is covered in an ECM (or sheath) that is synthesized at the slug tip forming a tube of proteins, glycoproteins and carbohydrates through which the cells comprising the slug move [21]. The sheath continues to be synthesized as the slug moves leaving behind a relatively cell-free trail of ECM that can be isolated and analyzed. Purified sheath contains CaM, full length CyrA, and the 45 kDa and 40 kDa CyrA cleavage products [28]. Coimmunoprecipitation studies showed that extCaM co-binds to CyrA as well as the 45kDa and 40kDa fragments in the ECM [27,28]. The binding of extCaM and CyrA represents another extracellular function for extCaM—binding to extracellular CaMBPs. Exactly how these proteins interact and function together remains to be elucidated. 

ExtCaM shows a gradient of diffuse distribution in the front and middle of the slug, but forms punctate deposits in increasing numbers further back in the slug and into the ECM trail [47]. In contrast, CyrA is most concentrated in the sheath at the tip of the slug diminishing in amounts towards the rear [27]. The localizations of extCaM and CyrA in the ECM surrounding the slug stage of development are both compelling and enigmatic because it could be important in the regulation of slug motility and morphogenesis during multicellular development. While the details remain to be elucidated, the binding of extCaM and CyrA has been suggested to have a developmental function in regulating the movement of cells within the slug [47]. 

As discussed above, the EGFL1 domain of CyrA increases cell motility and chemotaxis. In contrast, extCaM binds to CyrA resulting in decreased proteolytic processing which is another function for extCaM—the regulation of CaMBP proteolysis. It is possible that the binding of extCaM to CyrA controls the release of EGFL1-containing 45kDa and 40kDa fragments to regulate the localized rates of cell movement in the slug. However until the specific role of each of the EGFL1-containing components (*i.e.*, CyrA, C45 and C40) is elucidated it is not possible to further clarify their importance in the movement of cells in the slug or their potential function in morphogenesis [47].

## 3. Conclusions

The study of CyrA has added new insight into the evolution of matricellular proteins and the function of EGFL repeats. It has also provided data revealing that CaM is a valid extracellular protein with diverse stage-specific functions. The cysteine-rich CyrA is not homologous to any mammalian protein but it does share certain defining characteristics with ECM proteins designated as matricellular proteins. The characterization of the function of one of its four tandem EGFL repeats (*i.e.*, EGFL1) has not only provided the first evidence for EGFL repeat function in a lower eukaryote, the similarity in its function as a regulator of cell motility and the use of similar signaling mechanisms are strongly reminiscent of matricellular protein function in mammals. CyrA is unique in another way in that it is the first identified matricellular CaMBP. In keeping with this extCaM is present in the ECM where it not only binds to CyrA but it also controls the release of C-terminal EGFL repeat-containing fragments from the protein. In total, the data prove not only that extCaM is present in the eukaryotic amoebozoan *Dictyostelium* but also that it mediates several processes during both growth and development. Coupled with the research carried out on plants and animals these results reveal that extCaM may be as functionally critical and evolutionarily ubiquitous as intracellular CaM. Further research in a diversity of species will enhance our understanding of the true value of extCaM in signal transduction and cell function.
